# Revised Stability Scales of the Postural Stability Index for Human Daily Activities

**DOI:** 10.3390/e22101188

**Published:** 2020-10-21

**Authors:** Yu Ping Chang, Bernard C. Jiang, Nurul Retno Nurwulan

**Affiliations:** 1 Department of Industrial Management, National Taiwan University of Science and Technology, Taipei City 10607, Taiwan; M10701205@mail.ntust.edu.tw (Y.P.C.); bcjiang@mail.ntust.edu.tw (B.C.J.); 2 Department of Industrial Engineering, Sampoerna University, Jakarta 12780, Indonesia

**Keywords:** postural stability index, multiscale entropy, ensemble empirical mode decomposition, intrinsic mode function

## Abstract

Evaluation of human postural stability is important to prevent falls. Recent studies have been carried out to develop postural stability evaluation in an attempt to fall prevention. The postural stability index (PSI) was proposed as a measure to evaluate the stability of human postures in performing daily activities. The objective of this study was to use the PSI in developing the stability scales for human daily activities. The current study used two open datasets collected from mobile devices. In addition, we also conducted three experiments to evaluate the effect of age, velocity, step counts, and devices on PSI values. The collected datasets were preprocessed using the ensemble empirical mode decomposition (EEMD), then the complexity index from each intrinsic mode function (IMF) was calculated using the multiscale entropy (MSE). From the evaluation, it can be concluded that the PSI can be applied to do daily monitoring of postural stability for both young and older adults, and the PSI is not affected by age. The revised stability scales developed in this current study can give better suggestions to users than the original one.

## 1. Introduction

The second leading cause of death from accidents among people over 65 years old in Taiwan is falling [1]. To the elderly, serious falling may lead to a long-term bedridden situation or death. Unstable movement such as in walking can be barely noticeable and it may develop into falls when the treatment is too late. Postural instability has been reported as highly related to the risk of falling [2,3,4]. Thus, human postural stability evaluation can give a better understanding of preventing falls. Postural stability is the consistent predictor for the risk of falls, and maintaining postural stability can be a way to avoid falls.

Past studies used various approaches to measure human postural stability evaluation, such as the center of pressure (COP) [5,6,7,8,9], entropy-based methods [7,8,10,11,12], and the Star Excursion Balance Test [13]. The COP measurement using a force platform is the most commonly used method to evaluate postural stability. The COP stabilogram metrics often used to determine the postural stability are mean displacement velocity, total excursion, sway area, and root mean square [7,8,9]. Compared to the COP stabilogram metrics, entropy-based methods such as approximate entropy [5], sample entropy [8], and multiscale entropy [7,10,11,12] could better describe the COP data since human physiological is nonlinear and nonstationary [10]. Despite its validity in detecting the instability in human postural control, the force platform is expensive, cumbersome, and impractical for human daily activity evaluation. Nowadays, accelerometer is becoming popular because it is not expensive and could be used outside of the laboratory. The acceleration data obtained from the wearable accelerometers can be analyzed to study postural stability.

Cui et al. (2014) introduced the step stability index (SSI) using acceleration data to distinguish the non-fallers from the fallers by calculating the standard deviation of the intrinsic mode functions (IMFs) obtained from decomposing the signal data using ensemble empirical mode decomposition (EEMD) [14]. Although the SSI could categorize fallers and non-fallers, it may not be applicable when the subjects are all non-fallers. Nurwulan et al. (2019) developed the postural stability index (PSI) using multiscale entropy (MSE) to distinguish different stability states in healthy subjects [10,11]. The PSI was proposed with the consideration that the postural stability evaluation should be used not only to group the fallers and non-fallers using the index. Postural stability evaluation would be more useful if it can be used to predict the likelihood of falling in the healthy subjects even before they gradually become fallers.

The PSI was developed by decomposing the obtained signal data using 8-modes EEMD. The signal data was collected from 20 healthy young and 20 healthy older adults. The subjects were asked to perform normal walking, walking with obstacles, and intentional falling with wearable accelerometers attached to their ankles and knees. The MSE was then used to calculate the complexity index (CI) of each IMF. The PSI value is calculated by dividing the CI of the IMF3 by the sum of the CI of six IMFs. The third IMF was chosen as the dominant IMF because the frequency of the IMF3 is in the range of walking frequency, which is between 1.4–2.5 Hz [10]. Based on its PSI value, the stability scales were determined for each subject. The category of the stability scales includes stable, fairly stable, unstable, and danger [10].

The PSI was constructed based on the experiments in the laboratory with activities namely normal walking, walking with obstacles, and intentional falling. In real life, humans do not only walk horizontally on a flat surface but also walking upstairs, walking downstairs, speed walking, etc. The application of the PSI into other human daily activities would be more beneficial for fall prevention. In addition, Nurwulan et al. (2019) used wearable accelerometers with sampling frequency was set to 20 Hz [10]. Although wearable accelerometers are usually more accurate, they do not provide a long-term solution for activity monitoring. Nowadays, accelerometers are built in various wearable devices, such as smartphones and smartwatches. Smartphone- and smartwatch-based accelerometers can do continuous activity monitoring. Past studies showed that smartphones are able to provide accurate results for simple activity recognition [15].

Nurwulan et al. (2019) developed the PSI by emphasizing the possibility of falling on subjects [10]. The PSI was built by quantifying the signal data of normal walking, walking with obstacles, and intentional falling. However, humans can fall even when the obstacles do not exist. Therefore, the range of the PSI value on original stability scales may be limited to the conditions of the obvious potential of falling. This study aimed to apply the PSI into various normal human daily activities. To test the applicability of the PSI, public domain datasets from Kaggle were used. This current study also revised the range of the stability scales by checking the distribution of stability status of the datasets, to give users more suitable suggestions of their postural stability. In addition, we also conducted experiments to evaluate the effect of subjects’ characteristics such as age, velocity, and step count per second on the PSI value. Our findings from this evaluation are expected to provide suggestions on how to improve postural stability.

The rest of this article is organized in the following way. Section 2 provides the materials and methods employed in this study. It explains datasets used in this study and the theoretical backgrounds of the PSI. In Section 3, the results and discussion of the calculation and the revised stability scales are explained. Finally, Section 4 concludes the paper.

## 2. Materials and Methods

### 2.1. Kaggle Datasets

#### 2.1.1. Kaggle 1 Dataset: Motion Sense Dataset Smartphone Sensor Data

In this study, we used the public datasets obtained from Kaggle [16] to evaluate the performance of the PSI. The first dataset (Kaggle 1) was collected from 24 subjects (14 M, 10 F) aged ranging from 18 to 46 years. The subjects were asked to perform human daily activities with the iPhone 6-based accelerometer was placed inside of the front pocket. The sampling rate of the smartphone-based accelerometer was set to 50 Hz and the CrowdSense app was used to capture the signal data. The current study used walking, jogging, walking downstairs, and walking upstairs for the analysis using the PSI.

#### 2.1.2. Kaggle 2 Dataset: Run or Walk

The second dataset (Kaggle 2) was collected from a subject performing two activities which include walking and jogging for 12 days. The activities were done from 30 June 2017 to 9 July 2017. The subject used an iOS device with an app named Data Collection to capture the signal data. The iOS device was worn on either the right or left wrist.

### 2.2. Data Preprocessing

Both of the two datasets contain three axes of acceleration data, which are the *x*-axis, *y*-axis, and *z*-axis. When people are doing activities, the vibration of the body has not only one direction. Thus, this study considered the three axes of acceleration data by calculating the Euclidean distance between the origin. The data point was built with the square root of the sum of the square of the *x*-axis, *y*-axis, and *z*-axis.

The apps used for collecting acceleration data start to collect data from the start button until the stop button. The subject pressed the start button, then put the device back to the sensor location of the body. After the recording has finished, the mobile device was taken out from the pocket to press the stop button. Cutting the data points is important to ensure that only the activities are being analyzed.

### 2.3. Postural Stability Index

#### 2.3.1. Ensemble Empirical Mode Decomposition

Huang et al. (1998) introduced the empirical mode decomposition (EMD) algorithm to decompose non-linear and non-stationary signals into intrinsic mode functions (IMFs) [17]. There are two conditions that must be satisfied to make a function that can be an IMF [18]. First is for a given data, the difference of the sum of the number of local maximum and local minimum from the number of zero-crossings should differ at most by one. The other condition is the average value of the upper envelope defined by the local maximum and the lower envelope defined by the local minimum should be zero. IMFs show the characteristics of different parts of the signals. The frequency of the first IMF is the highest, and the later ones are lower [18].

When executing the EMD algorithm, the mode mixing problem will occur due to the signal intermittence. This mode mixing problem could cause the same IMF to contain different scale signals, or the same scale signal is assigned to different IMFs. In order to overcome this problem, Wu and Huang (2009) introduced the ensemble empirical mode decomposition (EEMD) by adding white noises $w_{i}\left(t\right)$ to original signal x(t) to eliminate the influence of the interference from noises [19].

(1) $$x_{i}\left(t\right)=x\left(t\right)+w_{i}\left(t\right)$$

Then, the signal $x_{i}\left(t\right)$ is decomposed into IMFs $c_{j}$ by extracting the $x_{i}\left(t\right)$ with the mean envelope $m_{k}\left(t\right)$. In the process of decomposing the IMFs, the residuals $r_{n}$ are the products after *n* number of IMFs are generated. The numbers of extrema *e* and zero-crossings of IMFs must be either equal or differ at most by one and the mean of local maxima and minima envelopes must be zero at any point [19].

(2) $$m_{k}\left(t\right)=\frac{e_{max}\left(t\right)-e_{min}\left(t\right)\;}{2}$$

(3) $$x_{i}\left(t\right)=\sum_{j=1}^{n}c_{j}+r_{n}$$

The processes in Equations (1) and (3) are reiterated for *m* trials, known as ensemble number, where a different white noise series $w_{i}\left(t\right)$ is added to the original signal in each trial. The final result of the EEMD process is obtained by averaging the total *n* IMFs related to *m* trials.

(4) $$IMF_{n}=\frac{1}{m}\sum_{i=1}^{m}IMF_{m,\;n}$$

A past study by Bari et al. [20] used a fast filtering technique to improve the performance of the EMD to limit the effect of the fast variations from the physiological signal data. In this study, 8-modes EEMD was chosen with the consideration that it could automatically identify the intrinsic time scales of the signal without assuming its stationary condition [10,11,14]. The standard deviation of 0.08 and ensemble numbers of 300, as suggested in the past studies, were used to decompose the acceleration data [10,11,21]. A comparative evaluation of the effect of the number of modes to the sensitivity of the calculation will be further discussed in Section 3.4.

#### 2.3.2. Multiscale Entropy

Entropy-based analysis has been widely applied to different fields, like medicine and biology because of its suitability in analyzing nonlinear data [7,8,10,11,12]. Multiscale entropy (MSE) was introduced to quantify the complexity of limited length time-series data [22]. For a given time series data with finite length, the MSE can be calculated by constructing coarse-grained time series. They are built by averaging a consecutively increasing number of the data points within non-overlapping windows.

(5) $$y_{i}^{\left(\Gamma \right)}i=\frac{1}{\Gamma }\overset{j\Gamma }{\underset{i=\left(j-1\right)\Gamma +1}{\sum }}x_{i},\;1\leq j\leq N/\Gamma$$

where Γ is the scale factor, and *N*/Γ is the length of each coarse-grained time series.

The Sample entropy (*SampEn*) for each coarse-grained time series is then plotted as a function of the scale factor.

(6) $$SampEn\left(m,\;r,\;N\right)\;=\;-\ln\left(\frac{A}{B}\right)$$

where *m* is the embedding dimension, r is the tolerance for accepting matches, N is the number of the data points, *A* is the number of template vector pairs which have Euclidian distance of $d\left[d_{m+1}\left(i\right),\;X_{m+1}\left(j\right)\right]<r\;$, and *B* is the number of template vector pairs which have Euclidian distance of $d\left[d_{m}\left(i\right),\;X_{m}\left(j\right)\right]<r\;$. Past studies evaluating the parameters of the *SampEn* suggested the values of *m* and *r* can be set as m=2 and r=0.15 [21,22].

The complexity index (*CI*) of each IMF is calculated by summing up the value of MSE for 20 scale factors (*sn*).

(7) $$CI=\overset{sn}{\underset{i}{\sum }}SampEn\left(i\right)$$

#### 2.3.3. Dominant IMF Determination

In the step stability index (SSI), the IMF4 was chosen as the dominant IMF due to the shape of the signal was similar to the original signal [14]. This visual method may be one of the methods of determining the dominant IMF. However, it may not be objective enough. The dominant IMF in the PSI method is the IMF3 because the instantaneous frequency of the IMF3 is within the range of walking frequency, which is 1.4–2.5 Hz [10,11,23]. Further, the Pearson’s correlation showed the IMF3 is correlated to gait variability [10]. Therefore, the PSI is formulated as the following.

(8) $$\mathrm{Stability}\;\mathrm{index}\;=\;\frac{CI\;of\;IMF3}{CI\;of\;IMF1\;+\;CI\;of\;IMF2\;+\;\ldots +\;CI\;of\;IMF6}$$

### 2.4. Revising the Stability Scales

The original stability scales are categorized into four states, they are stable, fairly stable, unstable, and danger states as the following [10].

80–100%: Stable
70–79%: Fairly stable, need minor attention
45–69%: Unstable, need high attention
<45%: Danger, cause falling

The PSI of all the activities from the two datasets (Kaggle 1 and Kaggle 2) were categorized into the original stability scales. Although walking is not a challenging activity for young adults, based on the original stability scales, walking is mostly categorized as danger. The distribution of the PSI values assigned to the stability scales might not be the same due to the differences in walking velocity, environments, and shoes worn by the users, or the degree of tightness of devices on the body. Thus, modification of the stability scales is important to make the PSI applicable for any human daily activities.

### 2.5. Factors Affecting the PSI Value

#### 2.5.1. Experiment 1: Age as the Factor

**Figure 1:**
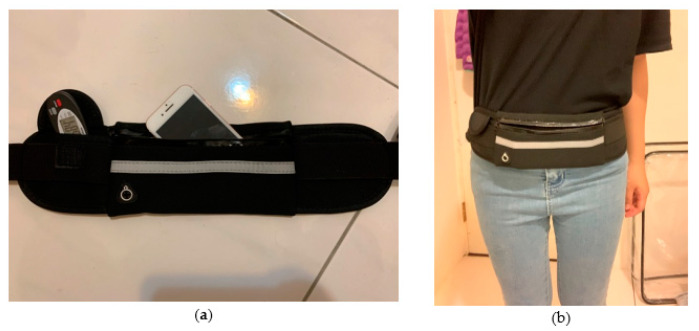
(**a**) The location of devices in the exercising fanny pack; (**b**) The subject with the devices.

In order to evaluate the factors affecting the PSI value, we conducted two experiments. The first experiment was conducted at Chung Yuan Cristian University’s sports field to see if age affects the PSI value. Three subjects were recruited in this experiment. Subject 1 was a 24-year-old female with a weight of 50 kg and a height of 155.5 cm, subject 2 was a 52-year-old female with a weight of 60 kg and height of 151 cm, and subject 3 was a 76-year-old male with a weight of 63 kg and height of 165 cm, all of them had no medical conditions. The female subjects were asked to walk with a distance of 100 m. This activity was done for seven days in January 2020. The male subject did two walking activities with a different velocity at a distance of 100 m. The walking activities were done for four days in March 2020. The iPhone Xs and pedometers were located on the waist (Figure 1) and the CrowdSense app was used for this experiment and the sampling rate was set to 50 Hz.

#### 2.5.2. Experiment 2: Velocity as the Factor

The second experiment was conducted at Beixin Elementary School’s sports field to see if the velocity and step counts would affect the PSI value. A 24-year old healthy female subject with a weight of 50 kg and a height of 155.5 cm was recruited in this experiment. The subject was asked to perform walking with low velocity, walking with high velocity, and jogging at a distance of 100 m. This activity was done for four days in March 2020. The iPhone Xs and pedometer were put on the waist of the subject. The sampling rate was also set to 50 Hz in this experiment.

## 3. Results and Discussion

### 3.1. Kaggle Datasets

#### 3.1.1. Kaggle 1 Dataset

In agreement with the past study [10], the frequency of IMF3 using Kaggle 1 dataset is within the range of walking frequency (1.4–2.5 Hz). Thus, the original PSI is applicable for this dataset. The stability scales were then built by doing the normalization as (stability index/80) × 100.

A paired sample t-test was used to evaluate whether the PSI can distinguish the activities. Using an alpha of 0.05, the PSI was able to discriminate walking and jogging (*p* < 0.001), waking downstairs and jogging (*p* < 0.001), and walking upstairs and jogging (*p* < 0.001) but failed to discriminate walking and walking downstairs (*p* = 0.947), walking and walking upstairs (*p* = 0.510), and walking downstairs and walking upstairs (*p* = 0.460). The original PSI was developed using obviously different activities such as walking, walking with obstacles, and intentional falling. However, the walking activities in Kaggle 1 dataset were all normal walking without any intervention. Thus, they are seen as similar activities based on original PSI.

The stability scales were then determined using the normalization of the PSI value. Using the stability scales, we can categorize the activities into the four stability states. Table 1 shows the number of subjects in each scale category for each activity. Based on the existing scales, most subjects are categorized in unstable and danger categories. Since the subjects in Kaggle 1 dataset were all young adults with flat shoes, walking should not be dangerous for them. Further, both walking and jogging are both either unstable and danger although the t-test showed that walking and jogging have significantly different PSI values. Therefore, it is important to revise the stability scales to be more applicable to human daily activities.

All subjects are in the danger category for jogging activity. This result may refer that the stability index and the stability scales are not suitable to evaluate jogging since they cannot distinguish its stability status for different subjects. The velocity of jogging is usually higher than walking and the difference in velocity may affect the stability index. This inference will be further analyzed using Kaggle 2 dataset.

#### 3.1.2. Kaggle 2 Dataset

Since the walking frequency is ranging from 1.4 to 2.5 Hz, it is evident that IMF1 is supposed to be the dominant IMF for this dataset. Thus, the PSI for Kaggle 2 dataset is CI of IMF1/(CI of IMF1 + CI of IMF2 + … + CI of IMF6). Table 2 shows the stability index and scales for walking and running.

Kaggle 2 dataset was collected from 1 subject performing walking and running on different days. Using the revised PSI, it can be seen from Table 2 that walking is categorized as stable. However, running is categorized as a dangerous activity. Although the PSI could distinguish walking and running in this dataset, the stability scales may need to be improved to fit the real situation.

### 3.2. Revising the Stability Scales

Improving the stability scales to fit the real situation is beneficial to get a better understanding of fall prevention. Since walking was categorized as “stable” in Kaggle 2 dataset but mostly as “danger” in Kaggle 1 dataset, we focused on Kaggle 1 dataset to revise the walking category of the scales. The range of “stable” status follows the original scales considering that the PSI values of the walking activities from the Kaggle 2 dataset were all above 80%. Thus, the “stable” range is still between 80–100% but this scale is renamed to “very stable”. Based on the PSI value from Kaggle 1 dataset, only two subjects are over 60%. The range of “fairly stable” status of original scales is between 70–79%. It is difficult for the subjects to reach this level, so the range is widened to 60–79%, and this level is renamed as “stable”. The “very stable” and “stable” statuses refer to the condition that the subjects are at ease when they are doing the activities.

Most subjects with “danger” status based on the original stability scales are on the scales of 40–45%. With regard to the fact that all subjects were young adults with flat shoes, it did not make sense if they were in danger only by walking in the campus area. Therefore, the third scale of 40–59% is named “fairly stable” which means that the subjects need to pay some attention to their postural stability.

The last two scales are revised based on the two higher-speed activities, which are jogging and running. The paired t-test of the PSI values of jogging (0.281 ± 0.040) from the Kaggle 1 dataset and running (0.192 ± 0.028) from the Kaggle 2 dataset showed they are significantly different (*p* < 0.001). The range of the fourth scale is set as 20–39% based on the PSI value jogging, and it is named as “unstable”. Whereas, the range of the last scale is below 20% and named as “danger”, which refers to the condition when the subjects have a high possibility of falling. In summary, the revised stability scales are as the following.

80–100%: Very stable (V).
60–79%: Stable (S).
40–59%: Fairly stable (F), need minor attention.
20–39%: Unstable (U), need high attention.
<20%: Danger (D), cause falling.

#### 3.2.1. Kaggle 1 Dataset

To evaluate our revised stability scales, we used the revised scales for Kaggle 1 and 2 datasets. Table 3 shows the comparison of original and revised stability scales for walking, jogging, walking downstairs, and walking upstairs. The original scales categorized most activities as either unstable or danger. While the revised scales categorized most subjects as fairly stable when walking and unstable when jogging. Since the PSI values of walking and jogging are significantly different (Table 1), the revised scales categorized the two activities better.

From Table 3, we can also see that the number of very stable and stable statuses in walking is more than the walking downstairs activity. However, the number of unstable walking downstairs is more than walking upstairs. This result does not seem to fit the real situation considering that walking upstairs should be more stable than walking downstairs. Walking downstairs is riskier than walking upstairs because of the acceleration added by gravity [24]. A past study by Chang Gung Medical Foundation also found that 10% of falling cases in the elderly in Taiwan were caused by walking upstairs and walking downstairs with more cases in walking downstairs [25].

#### 3.2.2. Kaggle 2 Dataset

Using Kaggle 2 dataset, we evaluated the categories of walking and running activities using the original and revised scales. Table 4 shows that the revised version does not affect the stability status of walking but the revised scales can differentiate the stability states of running. Thus, it is evident that the revised version can evaluate the stability states more precisely. However, this evaluation is only using the data from one subject, further study with a proper number of subjects is needed to validate this finding.

### 3.3. Factors Affecting the PSI Value

#### 3.3.1. Age Difference

In order to evaluate the factors affecting the PSI value, we analyzed the data obtained from our experiments. Based on the walking frequency of each IMF in experiment 1 dataset, both IMF2 and IMF3 are within the walking frequency of 1.4–2.5 Hz. However, the PSI value with IMF3 as the dominant IMF resulted in very low PSI for each subject. Thus, to make the PSI fits the real situation, we used IMF2 as the dominant IMF, and the PSI here was calculated as CI of IMF2/(CI of IMF1 + CI of IMF2 + … + CI of IMF6).

Table 5 shows that the older female subject walked more stable than the younger subject. Further, the elderly subject also walked more stable than the youngest subject. Intuitively, the younger subject should have better postural stability. Thus, there must be other factors affecting the PSI value such as velocity and step counts per second. Using the experiment 1 data, paired t-test evaluating the average velocity showed that jogging (0.281 ± 0.040) and running (0.192 ± 0.028) are significantly different (*p* < 0.001). However, based on the average step counts per second, jogging (1.864 ± 0.133) and running (1.914 ± 0.115) are not different (*p* = 0.470). Similarly, the age does not affect jogging and running (*p* = 0.02). With regard to these results, it can be said that there might still other factors that affect the PSI value such as different postures and conditions of the body.

Analysis and comparison of the PSI value for different subjects are difficult considering that the biological system is complicated. It is not the case if we analyze the PSI in the same subject. For example, in Table 6 we can see that the youngest subject performed the task at the same duration, but different step counts on 1/22 and 1/29. This means that more step counts lead to higher PSI value. The same trend is seen at 1/23 and 1/25, also 1/28 and 1/30 which refers that if the difference in step counts is large enough, the PSI value will be different although the duration is roughly the same. The elder subject also showed a similar trend where the low velocity walking on 3/21 and 3/23 have the same duration but the walking on 3/21 had more step counts and higher PSI value. This inference will be further discussed using experiment 2 in the next section.

#### 3.3.2. Velocity and Step Counts Differences

**Figure 2:**
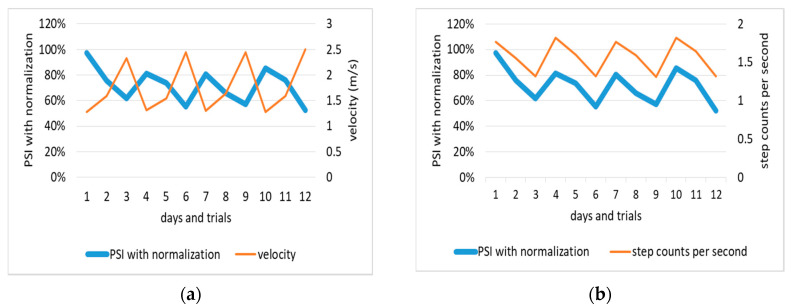
(**a**) Correlation of normalized PSI and velocity; (**b**) Correlation of normalized PSI and step counts.

Based on the frequency of the experiment two dataset, the IMF2 is determined as the dominant IMF, so the PSI is CI of IMF2/(CI of IMF1 + CI of IMF2 + … + CI of IMF6). Figure 2 shows that the activity with higher speed leads to a lower PSI value. On the contrary, the higher the step counts per second the higher the PSI value. Pearson’s correlation shows that the PSI value is affected by velocity (R = 0.921) and step counts per second (R = 0.911). This means the higher speed and longer step length the more unstable posture. For subjects detected with unstable walking, they can improve their postural stability by reducing the walking velocity or step with shorter step length.

### 3.4. Sensitivity Analysis of the PSI

Walking downstairs and walking upstairs have similar postural stability states based on the calculation using the PSI. This similarity does not fit the real situation since walking downstairs is usually more unstable. Thus, the adjusted stability index and scales are important to get a more accurate analysis. The adjustment can be done by choosing the dominant IMF based on its frequency. For example, the walking frequency is ranging from 1.4 to 2.5 Hz, so the dominant IMF should be the one with the frequency within the range. In the Kaggle 1 dataset, the frequency of walking downstairs and walking upstairs may be different. Using paired *t*-test, we found that walking downstairs and walking upstairs are significantly different (*p* = 0.015) when the IMF4 is chosen as the dominant IMF. Evaluating the frequency of each IMF before calculating the PSI might be an option. However, this is bothersome since we might need to change the dominant IMF before each analysis. Thus, we also evaluated the impact of using different parameters into the sensitivity of the analysis. Table 6 presents the paired t-test evaluation of the activities in the Kaggle 1 dataset using 8, 9, 10, 11, and 12 modes of EEMD with number scales of the MSE is set to either 10 or 20 scales. The PSI is using eight modes of EEMD, the results of the decomposition include raw signal data, 6 IMFs, and eight residual. After the decomposition, the complexity index of each IMF is calculated using 20 scale factors. Based on the comparison in Table 6, it can be seen that IMF4 as the dominant IMF could distinguish all activities when using nine modes of EEMD (7 IMFs) and 20 scale factors of MSE. Therefore, it concluded that the suggested revised PSI is the following.

$$Revised\;PSI=\;\frac{CI\;of\;IMF4}{CI\;of\;IMF1\;+\;CI\;of\;IMF2\;+\;\ldots +\;CI\;of\;IMF7}$$

Past studies reported that the MSE has limitations that could reduce the complexity evaluation in activities with subtle differences [26]. In order to overcome the drawbacks such as the reduction in artificial MSE due to the coarse-graining procedure and spurious MSE oscillations due to the suboptimal procedure for the elimination of the fast temporal scales. The refined multiscale entropy (RMSE) can overcome the drawbacks of the MSE by replacing the finite impulse response (FIR) filter with a low-pass Butterworth filter and using ***r*** as a function of the scale factor ***τ*** [26]. In our study, we used the EEMD to replace the filter process and from Table 6 above, it can be seen that the revised PSI with IMF4 as the dominant IMF could distinguish all daily activities. Further studies employing the RMSE after the decomposition process using 9 modes of EEMD might give a better explanation on this issue.

### 3.5. Different Mobile Devices Affect the PSI Value

**Figure 3:**
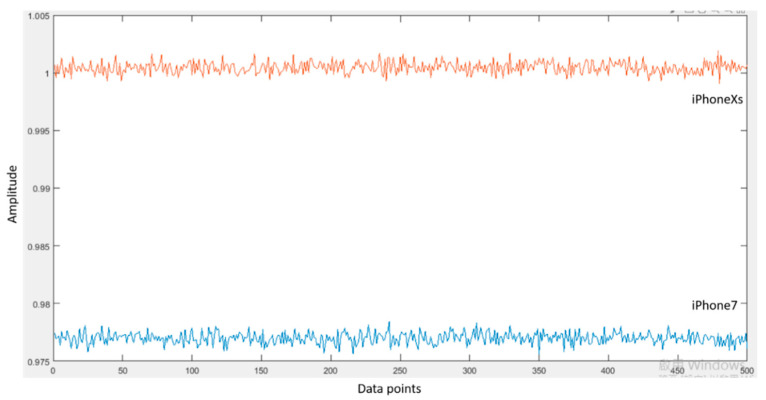
Comparison of iPhone 7 and iPhone Xs signal data.

**Figure 4:**
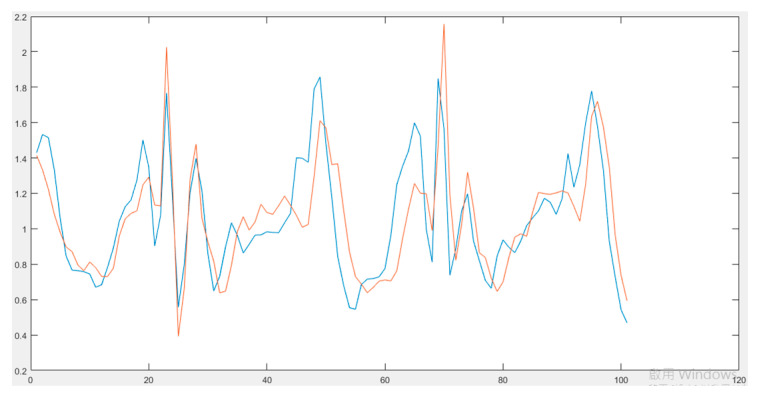
Comparison of shifted iPhone 7 (blue) and iPhone Xs (red) signal data.

In order to see whether different mobile devices affect the PSI value, we also conducted an experiment using two mobile devices at the same time. A 24-year-old female subject was asked to walk straight along a 160 m sidewalk with two smartphones (iPhone 7 and iPhone Xs) and a pedometer inside of the pocket for four days in May 2020. Table 7 shows the comparison of the stability index and scales of the walking activity captured by the iPhone 7 and iPhone Xs. From the comparison, it can be seen that iPhone Xs gave a more stable category for the walking activity than the iPhone 7. This might happen because of different built-in accelerometers inside of the different types of iPhone. We put both iPhones on a desk to see whether they give different signal data when there is no dynamic activity. From the comparison of raw signal data obtained from the iPhone 7 and iPhone Xs (Figure 3), iPhone 7 gave lower values of acceleration than the iPhone Xs. The effect of these different values of acceleration can be minimized by normalizing the raw signal data, this could be done by shifting the raw signal data of the iPhone 7 near to the iPhone Xs’ signal (Figure 4). However, even after this normalization, the signal data of two different types of iPhones are not similar. The iPhone 7 produced signal with more variation than the iPhone Xs. This variation could be caused by noises that in the end resulted in the less stable categorization of the activities. Other than the type of mobile device, the sampling frequency can also affect the PSI value. In this study, the sampling rate of the iPhone was set to 50 Hz. The original PSI used a wearable sensor with a sampling rate of 30 Hz. Future studies evaluating the effect of sampling rate on PSI value could improve the accuracy of the stability index and scales.

## 4. Conclusions

Postural stability index (PSI) was proposed to distinguish different postural stability states of healthy subjects. However, the PSI was developed using walking, walking with obstacles, and intentional falling. The above activities may not be applicable to the real situation since we never know when we will walk through obstacles or when we will fall. The current study used real human daily activities such as walking, walking upstairs, walking downstairs, and jogging to evaluate whether the PSI can be used to distinguish postural stability in a real situation. The paired t-test showed that the PSI is indeed able to discriminate against the activities. However, the stability scales of the original PSI cannot categorize the activities correctly. This current study revised the stability index and scales to better present the real-life situation. The original PSI used the IMF3 as the dominant IMF. However, this might not be applicable to other activities such as jogging and running. The sensitivity analysis comparing several modes of EEMD showed that using nine modes of EEMD with IMF4 as the dominant IMF could distinguish all activities, even the slightly different activities such as walking downstairs and walking upstairs. In addition, we also found that higher speed and fewer step counts lead to lower PSI value. Thus, in order to have stable movement, we need to lower the speed and reduce the step length. Although our revised PSI can differentiate the stability states of the daily activities, it could not discriminate against the differences caused by age. This may be caused by the number of subjects is not adequate. Future studies evaluating this matter with an adequate number of subjects might be able to explain further about this phenomenon.

## Figures and Tables

**Table 1:** Stability states of Kaggle 1 dataset.

Scales	Walking	Jogging	Walking Downstairs	Walking Upstairs
Stable	0	0	0	1
Fairly stable	1	0	0	1
Unstable	6	0	12	9
Danger	17	24	12	13

**Table 2:** Stability index and scales for walking and running.

Date	Stability Index	Normalization	Stability Scales
Walking			
6/30/2017	0.751	94%	Stable
7/1/2017	0.898	112%	Stable
7/6/2017	0.742	93%	Stable
7/7/2017	0.697	87%	Stable
7/10/2017	0.778	97%	Stable
7/16/2017	0.836	105%	Stable
7/17/2017	0.752	94%	Stable
Running			
6/30/2017	0.153	19%	Danger
7/2/2017	0.144	18%	Danger
7/4/2017	0.122	15%	Danger
7/6/2017	0.177	22%	Danger
7/9/2017	0.154	19%	Danger
7/11/2017	0.191	24%	Danger
7/15/2017	0.157	20%	Danger
7/17/2017	0.132	17%	Danger

**Table 3:** Comparison of original and revised stability scales for Kaggle 1 dataset.

Activity	Stability Scales					
Walking	Original	Stable	Fairly stable	Unstable	Danger	-
	Counts	0	1	6	17	-
	Revised	Very stable	Stable	Fairly stable	Unstable	Danger
	Counts	0	2	18	4	0
Jogging	Original	Stable	Fairly stable	Unstable	Danger	-
	Counts	0	0	0	24	-
	Revised	Very stable	Stable	Fairly stable	Unstable	Danger
	Counts	0	0	0	24	0
Walking downstairs	Original	Stable	Fairly stable	Unstable	Danger	-
	Counts	0	0	12	12	-
	Revised	Very stable	Stable	Fairly stable	Unstable	Danger
	Counts	0	1	16	7	0
Walking upstairs	Original	Stable	Fairly stable	Unstable	Danger	-
	Counts	1	1	9	13	-
	Revised	Very stable	Stable	Fairly stable	Unstable	Danger
	Counts	1	5	9	9	0

**Table 4:** Comparison of original and revised stability scales for Kaggle 2 dataset.

Date	Normalized Stability Index	Original Scales	Revised Scales
Walking			
6/30/2017	94%	Stable	Very stable
7/1/2017	112%	Stable	Very stable
7/6/2017	93%	Stable	Very stable
7/7/2017	87%	Stable	Very stable
7/10/2017	97%	Stable	Very stable
7/16/2017	105%	Stable	Very stable
7/17/2017	94%	Stable	Very stable
Running			
6/30/2017	19%	Danger	Danger
7/2/2017	18%	Danger	Danger
7/4/2017	15%	Danger	Danger
7/6/2017	22%	Danger	Unstable
7/9/2017	19%	Danger	Danger
7/11/2017	24%	Danger	Unstable
7/15/2017	20%	Danger	Danger
7/17/2017	17%	Danger	Danger

**Table 5:** Stability index and scales for the walking activity of the female subjects.

Date	Remark	Normalized Stability Index	Revised Stability Scales	Step Counts	Duration (s)
24-year-old female					
1/22		72%	Stable	188	95
1/23		75%	Stable	134	84
1/25		95%	Very stable	167	86
1/26		87%	Very stable	168	87
1/28		68%	Stable	167	90
1/29		48%	Fairly stable	171	95
1/30		94%	Stable	177	91
52-year-old female					
1/22		86%	Very stable	204	99
1/23		94%	Very stable	172	89
1/25		106%	Very stable	161	85
1/26		117%	Very stable	163	95
1/28		99%	Very stable	166	87
1/29		84%	Very stable	174	94
1/30		96%	Very stable	181	89
76-year-old male					
3/21	Slow	82%	Very stable	172	89
	Fast	70%	Stable	155	79
3/22	Slow	62%	Stable	160	85
	Fast	78%	Stable	148	72
3/23	Slow	78%	Stable	165	88
	Fast	77%	Stable	149	73

**Table 6:** Sensitivity analysis of IMF3 and IMF4 as the dominant IMF.

Parameters	Walking-Jogging	Walking-Downstairs	Walking-Upstairs	Jogging-Downstairs	Jogging-Upstairs	Downstairs-Upstairs
IMF3 as the dominant IMF						
8 modes, 20 scales	*p* < 0.001	0.993	0.494	*p* < 0.001	*p* < 0.001	0.471
9 modes, 20 scales	*p* < 0.001	0.859	0.494	*p* < 0.001	*p* < 0.001	0.551
10 modes, 20 scales	*p* < 0.001	0.733	0.504	*p* < 0.001	*p* < 0.001	0.636
11 modes, 20 scales	*p* < 0.001	0.099	0.618	*p* < 0.001	0.002	0.635
12 modes, 20 scales	0.002	0.018	0.609	*p* < 0.001	0.004	0.446
8 modes, 10 scales	*p* < 0.001	0.086	0.112	*p* < 0.001	*p* < 0.001	0.523
12 modes, 10 scales	*p* < 0.001			*p* < 0.001	*p* < 0.001	
IMF4 as the dominant IMF						
8 modes, 20 scales	*p* < 0.001	0.003	0.179	*p* < 0.001	*p* < 0.001	0.016
9 modes, 20 scales	*p* < 0.001	0.003	0.043	*p* < 0.001	*p* < 0.001	0.03
10 modes, 20 scales	*p* < 0.001	0.003	0.01	*p* < 0.001	*p* < 0.001	0.078
11 modes, 20 scales	*p* < 0.001	0.007	0.016	*p* < 0.001	*p* < 0.001	0.182
12 modes, 20 scales	*p* < 0.001	0.017	0.012	*p* < 0.001	*p* < 0.001	0.687
8 modes, 10 scales	*p* < 0.001	0.004	0.263	*p* < 0.001	*p* < 0.001	0.022
12 modes, 10 scales	*p* < 0.001	0.149	0.786	*p* < 0.001	*p* < 0.001	0.149

**Table 7:** Stability index and scales for walking activity with the iPhone Xs and iPhone 7.

Date	Trial	Normalized Stability Index		Revised Stability Scales		Step Counts	Distance (m)	Duration (s)
		iPhone Xs	iPhone 7	iPhone Xs	iPhone 7			
5/12	1	58%	59%	Fairly stable	Fairly stable	267	160	134
5/12	2	74%	49%	Stable	Fairly stable	266	160	143
5/14	1	66%	57%	Stable	Fairly stable	282	160	139
5/14	2	65%	53%	Stable	Fairly stable	271	160	146
5/16	1	69%	62%	Stable	Stable	249	160	134
5/16	2	62%	53%	Stable	Fairly stable	260	160	143
5/19	1	73%	59%	Stable	Fairly stable	260	160	139
5/19	2	70%	56%	Very stable	Fairly stable	255	160	139
